# Editorial: The public health problem of burnout in health professionals

**DOI:** 10.3389/fpubh.2023.1173312

**Published:** 2023-03-21

**Authors:** Alexander Hodkinson, David Peters, Maria Panagioti, Josep Pifarre, Oriol Yuguero

**Affiliations:** ^1^Division of Population Health, Health Services Research & Primary Care, The University of Manchester, Manchester, United Kingdom; ^2^Centre for Resilience, University of Westminster, London, United Kingdom; ^3^Medicine and Surgery Department, Universitat de Lleida, Lleida, Spain; ^4^ErLab, Lleida Institute for Biomedical Research IRBLLEIDA, Lleida, Spain

**Keywords:** burnout, health profession, pandemic, global health, ethics

## Introduction

With this title we launch a Research Topic to talk about burnout among health professionals. With rising levels of burnout it has become a veritable public health problem, and of growing concern not only to healthcare staff and occupational health specialists.

I would like to thank the team of editors who have participated in this Research Topic, Dr. David Peters, of the University of Westminster, Dr. Josep Pifarré of the University of Lleida, and Dr. Maria Panagioti and Dr. Alexander Hodkinson, from the University of Manchester and, of course, the more than 100 authors who have contributed their manuscripts to our Research Topic. We all share their desire to shed light on the complex matter of burnout among healthcare practitioners.

Since Maslach carried out her first studies on health professional burnout and published the Maslach Burnout Inventory in 1981, a multitude of research discussions and studies (1) have been published. In 2017, Shanafelt and Noseworthy (2) proposed organizational strategies and policies to help reduce burnout among healthcare professionals in a widely cited study. In this Research Topic we have seen that the concept of burnout sometimes overlaps with compassion fatigue or post-traumatic stress in the face of adverse circumstances. Moreover, actually the controversy about burnout include a large overlap with depression too.

Around the world, the spiraling demands on healthcare systems during the SARS-CoV-2 pandemic pushed health professionals to the limit, especially younger professionals and those on the frontline of care. It also revealed that in many countries the health and care systems were sustained only thanks to the dedication, vocation and overwork of their professionals. In response, we proposed this Research Topic to try to provide evidence-based answers for combating the global burnout epidemic now affecting thousands of health professionals.

The main aim of this Research Topic is to showcase research currently being conducted on burnout among health professionals, as an occupational health and public health problem, and various reviews (3) and articles have been published in recent years presenting different proposals and recommendations for burnout prevention and mitigation.

## Burnout, a global problem

One of the key conclusions of our Research Topic is that although burnout affects health professionals around the world, some groups such as emergency professionals and primary care professionals may be affected more adversely. Thus, another objective is to ascertain the variations across different countries. The 16 articles submitted have provided us with evidence from teams in China, Africa, South America and Europe. In the face of this global pandemic the World Health Organization has promoted the recognition of burnout as an occupational illness by incorporating it into the international classification of diseases (ICD-11) (4).

In many countries, where health professionals have to perform non-healthcare-related tasks, a lack of job satisfaction may contribute to low professional realization, one of Maslach's underlying causes of burnout. We know too that professionals' lack of alignment with the values of their employer and reduced involvement in their work plan are strongly related to burnout.

## Proposals to reduce burnout

Burnout is a complex problem that cannot be addressed only by financial input alone, but requires health care organizations to commit to, and invest in its prevention in recognition of a deeper need to promote healthy work environments and co-produce organizational improvements in partnership with health professionals. Health professionals' alignment with the values and mission of their non-clinical employer can be enhanced with the recognition of the important work done on a daily basis, by managers and leaders. The active participation of clinicians and front-line professionals in making management decisions that may affect them is another an essential aspect in the prevention of burnout. Moreover, there is a need to challenge the culture of workaholism still so prevalent among those who enter health care but which, over time can invite burnout.

On the bright side, this issue presents factors that may protect health care professionals from burnout. For example, we can learn from the skills of those professionals who continue to perform enthusiastically in their daily practice, and perhaps surprisingly from first-generation immigrant professionals. At present many health professionals choose to migrate between countries, and there is evidence to suggest that new immigrant professionals present lesser degrees of burnout.

Although we highlighted the importance of organizational improvements, individual psychological interventions such as mindfulness have proven to be effective in reducing burnout and improving the wellbeing of professionals and medical students. They should be also promoted under time-protected schemes across health care organizations.

## Conclusions

With this Research Topic we conclude that burnout is a global problem of all health care systems, affecting almost all professional categories, including students of health sciences.

We need to uncover and widely implement a work culture that promotes job satisfaction and active participation in decision-making among health professionals. Finally, health organizations and institutions play a fundamental role in promoting initiatives for enhancing the emotional wellbeing of their workers and appointing leaders (5) who share the view that health professionals are the cornerstone of health care systems everywhere.

## Author contributions

All authors listed have made a substantial, direct, and intellectual contribution to the work and approved it for publication.
